# Revisional bariatric surgery after adjustable gastric band: a multicenter Polish Revision Obesity Surgery Study (PROSS)

**DOI:** 10.1186/s12893-023-02002-w

**Published:** 2023-04-20

**Authors:** Natalia Dowgiałło-Gornowicz, Michał Janik, Paweł Lech, Grzegorz Kowalski, Piotr Major, PROSS –Collaborative Study Group, Michał Pędziwiatr, Justyna Rymarowicz, Piotr Zarzycki, Tomasz Stefura, Karol Ciszek, Piotr Małczak, Piotr Myśliwiec, Hady Razak Hady, Paulina Głuszyńska, Monika Proczko-Stepaniak, Michał Szymański, Maciej Walędziak, Andrzej Kwiatkowski, Magdalena Materlak, Katarzyna Bartosiak, Łukasz Czyżykowski, Maciej Mawlichanów, Piotr Kowalewski, Jacek Szeliga, Wojciech Kupczyk, Anna Harań, Rafał Mulek, Michał Krefft, Michał Wysocki, Michał Orłowski, Paula Franczak, Artur Binda, Wiesław Tarnowski, Paweł Jaworski, Mateusz Kamiński, Maciej Pastuszka, Wojciech Lisik, Paweł Szymański, Bartosz Katkowski, Michał Leśniak, Michał Łabul

**Affiliations:** 1 Department of General, Minimally Invasive and Elderly Surgery, Collegium Medicum, University of Warmia and Mazury, Olsztyn, Poland; 2 Department of Surgery, Military Institute of Aviation Medicine, Warsaw, Poland; 3 Surgery Clinic Mazan, Katowice, Poland; 4 2nd Department of General Surgery, Jagiellonian University Medical College, Cracow, Poland

**Keywords:** Revisional bariatric surgery, Adjustable gastric band, Bariatric surgery, Revision surgery, AGB

## Abstract

**Background:**

Adjustable gastric band (AGB) hadbeen the preferred treatment for morbid obesity because it is minimally invasive and reversible. But now it seems to be slowly becoming a historic procedure due to the disappointing effects. The aim of the study was to systematize and present the available data on revisional bariatric surgery (RBS) after AGB among Polish patients.

**Methods:**

It is a multicenter, retrospective analysis of patients undergoing laparoscopic RBS after AGB in 12 Polish bariatric centers. The database included patient demographics, comorbidities and surgical outcomes.

**Results:**

The group consisted of 234 patients who underwent AGB, which accounted for 29% of revisional cases recorded in the Polish Revisional Obesity Surgery Study (PROSS). 195 were women (83%), and 39 were men (17%). One hundred seventy-five patients after AGB experienced a weight regain (74.5%), 36 patients a gastric band slippage (15.0%), 14 patients had gastric band intolerance (6.0%). Types of RBS included 116 sleeve gastrectomies (SG) (49.4%), 86 Roux-en Y gastric by-passes (RYGB) (36.6%), 20 one anastomosis gastric by-passes (OAGB) (8,5%). The highest weight loss expressed as %EBMIL was observed after OAGB (63.5 ± 32.4%).

**Conclusions:**

The main indication for RBS after AGB was weight regain. SG was the most frequently chosen type of RBS after AGB. RBS after AGB leads to weight loss and improvement in type 2 diabetes and hypertension with an acceptable low risk of complications.

**Trial registration:**

NCT05108532.

## Background

Obesity is a growing problem worldwide and the number of bariatric surgeries continues to increase [1]. Currently, there are more than 20 types of procedures tailored to the obese patient [2]. Not every method of surgical treatment of obesity has turned out to be as effective as originally thought and planned. Following the widespread use of laparoscopy in bariatric surgery, the laparoscopic adjustable gastric band (AGB) had become one of the most performed procedures. This was due to its short operative time, relatively easy to perform, and preliminary results were promising [1].

AGB -was the preferred treatment for morbid obesity because it was minimally invasive and reversible. This led to many surgeries. But now it seems to be slowly becoming a historic procedure due to the disappointing effects of weight loss and weight regain [3]. Currently, thousands of patients require bariatric revision due to unsatisfactory metabolic outcomes or postoperative complications after AGB [3, 4]. The band removal procedure is relatively easy and willingly performed by many bariatric surgeons. The problem is the type of revisional bariatric surgery (RBS) that should be performed on the patient. It often depends on the reason of the reoperation or the habits of the surgeons. The aim of the study was to systematize and present the available data on RBS after AGB among Polish patients.

## Methods

### Study design

It is a multicenter, retrospective, non-randomised analysis of the collected data between 2019 and 2020 under the patronage of Metabolic and Bariatric Surgery Chapter and Videosurgery Chapter of the Association of Polish Surgeons. In each department, authors involved in the surgical treatment of obesity entered data on bariatric patients undergoing laparoscopic RBS to build a comprehensive database. The inclusion criteria for this study met the bariatric surgery eligibility criteria [5]. In the absence of overarching guidelines for patient eligibility for RBS, each center has its own criteria. Patients with missing or inconsistent data were excluded from the study.

The database included patient demographics: sex, age, maximum body weight, weight before primary surgery, weight before RBS, height, and body mass index (BMI). It also contained information on comorbidities in patients: type 2 diabetes (T2D) and hypertension (HT) and their remission according to standardized outcomes reporting [6]. Additionally, it included data on AGB (length of hospital stay (LOS), complications, outcomes) and data on RBS (indications for RBS, LOS, type of surgery, complications, outcomes). Recurrence of obesity was defined as a regain of weight after initial successful weight loss (defined as percentage of excess weight loss (EWL%) > 50%) or a failure to achieved a successful weigh loss [6].

### Surgical technique and perioperative care

Surgical technique and protocol of perioperative care, including preoperative, intraoperative, and postoperative interventions, were standard at each participating center. Patients were treated by a multidisciplinary team of surgeons, physicians, nurses, dieticians, and psychologists at each bariatric center.

### Statistical analysis

The analysis was performed using SAS® On Demand for Academics software (SAS Institute, Cary, NC, USA). Continuous variables were expressed using mean and standard deviation. Categorical variables were presented using percentage. For analysis of variance, general linear model was used. Categorical variables were compared using Fisher's exact tests. Statistical significance was set at *p* < 0.05.

### Ethical considerations

The data were completely anonymized. The study was conducted in accordance with the ethical standards of Helsinki Declaration of 1964 and its subsequent amendments (Fortaleza). The protocol was registered at clinical trials.gov (NCT05108532, 05/11/2021). The study was closely monitored by the principal investigator who processed and verified any missing or unclear data submitted to the central database. The study was approved by the Bioethics Committee of the Regional Chamber of Physicians, District of Warmia and Mazury, Poland (23/2021/VIII).

## Results

### Study group characteristics

The group consisted of 234 patients who underwent AGB, which accounted for 29% of revisional cases recorded in the Polish Revisional Obesity Surgery Study (PROSS). 195 were women (83%), and 39 were men (17%). The mean age was 47.7 ± 9.7 years. Seventy-six patients had HT and 13% had T2D. Alcohol consumption was reported in 32% and 17% reported smoking prior to bariatric treatment. 18% of patients used NSAIDs more than once a week. The mean follow-up time was 23.0 ± 29.6 months, Table 1.

**Table 1:** Demographic characteristics for analyzed patients

Characteristics	*N* = 234
	Mean (SD) or %
Age (years)	47.7 ± 9.7
Sex, female (%)	83%
Maximum BMI (kg/m2)	45.1 ± 6.8
BMI before bariatric surgery (kg/m2)	43.9 ± 6.6
Lowest BMI after primary surgery (kg/m2)	33.2 ± 7.4
BMI before revisional surgery (kg/m2)	40.6 ± 6.9
BMI after revisional surgery (kg/m2)	32.4 ± 5.8
Hypertension	32%
Type 2 diabetes	13%
NSAIDs users	18%
Former Smoker	17%
Former alcohol consumption	32%
Interval time between primary and revisional time, years	5.7 ± 3.7
Follow up time (months)	23.0 ± 29.6
Type of revisional bariatric surgery	
Sleeve gastrectomy, SG	49.4%
Roux-en Y gastric bypass, RYGB	36.6%
One anastomosis gastric by-pass, OAGB	8.5%
Adjustable Gastric Band removal, AGB removal	2.5%
Subsequent Adjustable Gastric Band, AGB	2.1%

### Indications for RBS

After AGB, 175 patients experienced a weight regain (74.5%), that met the bariatric criteria, 36 patients experienced a gastric band slippage (15.0%), 14 patients had gastric band intolerance (6.0%), 7 patients developed gastric outlet obstruction (3.0%). Two patients had gastric band migration (1%) and one patient had esophageal perforation (0.5%), Table 2.

**Table 2:** Indication for revisional bariatric surgery

Indication	Number (%)
Weight regain	175 (74.5%)
Gastric band slippage	36 (15.0%)
Gastric band intolerance	14 (6.0%)
Gastric outlet obstruction	7 (3.0%)
Gastric band migration	2 (1.0%)
Oesophagus perforation	1 (0.5%)

### Types of RBS

Types of RBS included 116 sleeve gastrectomies (SG) (49.4%), 86 Roux-en Y gastric by-passes (RYGB) (36.6%), 20 one anastomosis gastric by-passes (OAGB) (8,5%), and other procedures (5.4%) Table 1.

### Weight loss

The mean BMI before AGB was 43.9 ± 6.6 kg/m2. The mean lowest BMI after AGB was 33.2 ± 7.4 kg/m2. Before RBS, the mean BMI was 40.6 ± 6.9 kg/m2. After RBS, the mean BMI was 32.4 ± 5.8 kg/m2 (Table 1). After AGB, the percentage of excess BMI loss (EBMIL%) and delta BMI (dBMI) were 15.39 ± 31.29 and 3.32 ± 5.77, respectively. EBMIL% and TWL% after RBS were 47.86 ± 59.48% and25.4 ± 12.9%. The highest weight loss expressed as %EBMIL and TWL% was observed after RBS in patients who underwent OAGB (63.5 ± 32.4% and 27.5 ± 11.4%, respectively). %EBMIL and TWL% in RYGB group were 53.2 ± 29.3% and 24.1 ± 12.2%. %EBMIL and TWL% in SG patients were: 52.6 ± 42.6% and 26.98 ± 13.2%. The lowest weight loss was observed in patients who underwent subsequent AGB—%EBMIL and TWL% were 41.9 ± 55.9% and 22.1 ± 17.8%, Table 3.

**Table 3:** Weight loss outcomes after revisional bariatric surgery

Outcome	Mean (SD)
dBMI [kg/m2]^*^	8.21 ± 6.25
Sleeve gastrectomy,SG	9.7 ± 5.7
Roux-en Y gastric bypass,RYGB	7.3 ± 5.2
One anastomosis gastric by-pass, OAGB	9.2 ± 5.7
Adjustable Gastric Band removal, AGB removal	-7.4 ± 7.3
Subsequent Adjustable Gastric Band	2.4 ± 3.2
EBMIL% [%]^*^	47.9 ± 59.5
Sleeve gastrectomy,SG	52.6 ± 42.6
Roux-en Y gastric bypass,RYGB	53.2 ± 29.3
One anastomosis gastric by-pass, OAGB	63.5 ± 32.4
Adjustable Gastric Band removal, AGB removal	-164.6 ± 201.7
Subsequent Adjustable Gastric Band, AGB	41.9 ± 55.9
TWL% [%]^*^	**25.4 ± 12.9**
*Sleeve gastrectomy,SG*	26.98 ± 13.2
*Roux-en Y gastric bypass,RYGB*	24.1 ± 12.2
*One anastomosis gastric by-pass, OAGB*	27.5 ± 11.4
*Adjustable Gastric Band removal, AGB removal*	8.7 ± 10.6
*Subsequent Adjustable Gastric Band, AGB*	22.1 ± 17.8

^*^Analysis of variance (GLM Procedure in SAS Studio) showed *p* < 0.05 for dBMI, EBMIL%, and TWL%

### Remission of obesity-related comorbidities

T2D was diagnosed in 31 patients (13.2%) before RBS. Follow-up data were available for 28 patients. After RBS, 8 patients (28.6%) achieved complete remission, 7.2% partial remission of T2D. Improvement in T2D was observed in the group of 8 patients. Preoperative HT was diagnosed in 76 patients (32.3%). Follow-up data were available for 59 patients. Complete remission of HT was observed in 31.7%. Improvement in HT was observed in 31.7%. No effect was found in 35%, Table 4.

**Table 4:** Changes in comorbidities after RBS

Type of RBS	LSG	RYGB	OAGB	AGB removal	Subsequent AGB
Type 2 diabetes*	*N* = 11	*N* = 5	*N* = 7	*N* = 3	*N* = 2
Complete remission	18.2%	20%	71.4%	0%	0%
Partial remission	0%	20%	14.3%	0%	0%
Improvement	36.4%	20%	14.3%	66.7%	0%
Without change	45.4%	40%	0%	33.3%	100%
Hypertension**	*N* = 31	*N* = 15	*N* = 7	*N* = 3	*N* = 4
Complete remission	32.3%	20%	28.6%	66.7%	50%
Improvement	35.5%	20%	71.4%	0%	0%
Without change	29%	60%	0%	33.3%	50%

^*^P-value in Fisher Test was 0.078

^**^P-value in Fisher Test was 0.055

### Complications and LOS after RBS

Complications after RBS are described in Table 5. The RBS with the highest complication rate was SG. 24.1% experienced leakage, bleeding, ileus or GERD. The median LOS was 3.2 days after RBS. The shortest for SG (2.9 days) and the longest after subsequent AGB (4.0 days), Table 5.

**Table 5:** Complications and LOS after RBS

Type of RBS	LSG	RYGB	OAGB	AGB removal	Subsequent AGB
	*N* = 116	*N* = 86	*N* = 20	*N* = 6	*N* = 5
Leakage*	2.6%	1.2%	0%	0%	0%
Bleeding*	0.9%	3.5%	0%	0%	0%
Ileus*	6.0%	3.5%	0%	0%	0%
GERD*	14.7%	1.2%	0%	0%	40%
None^a^*	75.9%	90.7%	100%	100%	60%
LOS (days)**	2.9 ± 1.2	3.7 ± 1.0	2.7 ± 1.1	3.7 ± 1.0	4.0 ± 1.0

^a^There was none of: leakage, bleeding, iledus ang GERD

^*^P-value in Fisher Test was < 0.05

^**^Analysis of variance (GLM Procedure in SAS Studio) showed *p* < 0.005 for LOS

## Discussion

Our study is a retrospective analysis of 234 patients undergoing RBS after AGB. This study includes data on the largest group of patients undergoing RBS in Poland, collected as a part of PROSS project [7]. According to an IFSO Survey, there was a huge drop in AGB’s performance from 42,3% to 1,4% between 2008 and 2018 [1]. Furthermore, a meta-analysis by Koh et al. reported that AGB is one of the most common bariatric surgeries requiring revision [8]. So we believe that our work will be a useful in clinical practice of RBS analysis.

Recent analysis showed that AGB has a high removal rate, up to 50% 15 years after surgery [3, 4, 9]. On the other hand, we can also find papers with lower rate. O’Bien et al. reported approximately 6% of band removal over 15 years of follow-up, but 76% of patients required surgical band replacement due to complications [10]. So the removal rate may vary depending on the management policy in the case of band failure. Nevertheless, all the papers found reported an exceptionally high rate of RBS requirement. This contributed to the drastic decline in the use of AGB [1].

The main cause of RBS in our study was a weight regain (74.5%) and secondly gastric band slippage and intolerance (15%, 6% respectively). The weight regain or insufficient weigh loss is also a main reason for band removal in other reports, but the proportion is not dominated by this [3, 10, 11]. The reason for this difference can be found in our study design. We analyzed not all the AGB inserted at this time, but only the cases requiring RBS, unlike the papers that examined the AGB in general [3, 10–12].

Almost half of the analyzed patients underwent SG as a revisional procedure, followed by RYGB and OAGB. We found various tendencies in the literature [3, 11–14]. Rafols et al. reported that three-quarters of their patients undergone RYGB as RBS, while Chansaroy et al. performed nearly half of OAGB [11, 12]. It can therefore be concluded that the choice of RBS depends on the experience and habits of a given center. It is also worth noting that OAGB gains over SG, which we find in the risk of GERD [1, 15]. According to the latest data, OAGB has a similar effect in reducing GERD symptoms as RYGB, evidenced by patient assessment of upper gastrointestinal disorder-symptom severity index (PAGI-SYM) standardized questionnaire, upper endoscopy, 24-h pH monitoring and manometry [16].

If weight regain is one of the main causes of band removal, weight loss after RBS is an especially important factor when choosing revisional surgery. In our study, OAGB achieved the best results expressed as %EBMIL, which was 63.5% at the time of follow-up, followed by RYGB and LSG, both around 53% each. Other authors also observed comparable results in favor of the OAGB [11, 12].

RBS after AGB can be performed in both a one-step and two-step procedure [17–20]. Unfortunately, our data cannot determine which type was selected. Nevertheless, a recent meta-analysis by Zadeh et al. showed that there does not appear to be a significant difference in the overall leak rate between one- and two-step AGB conversions [21]. In our study, the perioperative complications requiring intervention were less than 3%. 4 patients (1.7%) experienced a leak, and 3 patients (1.3%) experienced postoperative bleeding.

The limitation of the study is its retrospective character. Some procedures were performed selectively, such as subsequent AGB, so their statistical comparison can be misleading. In addition, we do not have a universal protocol for revisional surgery, therefore the indications and the choice of procedure may differ depending on the center.

## Conclusions

The main indication for RBS after AGB was weight regain. SG was the most frequently chosen type of RBS after AGB. RBS after AGB leads to weight loss and improvement in T2D and HT with an acceptable low risk of complications.

## Data Availability

The datasets used and/or analysed during the current study are available from the corresponding author on reasonable request.

## Abbreviations

AGB: AGB adjustable gastric band
RBS: RBS revisional bariatric surgery
PROSS: Polish Revisional Obesity Surgery Study
SG: Sleeve gastrectomy
RYGB: Roux-en Y gastric by-pass
OAGB: One anastomosis gastric bypass
T2D: Type 2 diabetes
HT: Hypertension
LOS: Length of hospital stay
BMI: Body mass index
EWL%: Percentage of excess weight loss
EBMIL%: Percentage of excess BMI loss
dBMI: Delta BMI
